# Forward Genetic Screening for Regulators Involved in Cholesterol Synthesis Using Validation-Based Insertional Mutagenesis

**DOI:** 10.1371/journal.pone.0112632

**Published:** 2014-11-26

**Authors:** Wei Jiang, Jing-Jie Tang, Hong-Hua Miao, Yu-Xiu Qu, Jie Qin, Jie Xu, Jinbo Yang, Bo-Liang Li, Bao-Liang Song

**Affiliations:** 1 Institute of Biochemistry and Cell Biology, Shanghai Institutes for Biological Sciences, Chinese Academy of Sciences, Shanghai, China; 2 College of Life Sciences, Wuhan University, Wuhan, China; 3 School of Life Sciences, Lanzhou University, Lanzhou, China; Institute of Molecular and Cell Biology, Biopolis, United States of America

## Abstract

Somatic cell genetics is a powerful approach for unraveling the regulatory mechanism of cholesterol metabolism. However, it is difficult to identify the mutant gene(s) due to cells are usually mutagenized chemically or physically. To identify important genes controlling cholesterol biosynthesis, an unbiased forward genetics approach named validation-based insertional mutagenesis (VBIM) system was used to isolate and characterize the 25-hydroxycholesterol (25-HC)-resistant and SR-12813-resisitant mutants. Here we report that five mutant cell lines were isolated. Among which, four sterol-resistant mutants either contain a truncated NH_2_-terminal domain of sterol regulatory element-binding protein (SREBP)-2 terminating at amino acids (aa) 400, or harbor an overexpressed SREBP cleavage-activating protein (SCAP). Besides, one SR-12813 resistant mutant was identified to contain a truncated COOH-terminal catalytic domain of 3-hydroxy-3-methylglutaryl-coenzyme A reductase (HMG-CoA reductase). This study demonstrates that the VBIM system can be a powerful tool to screen novel regulatory genes in cholesterol biosynthesis.

## Introduction

The feedback regulation of cholesterol synthesis in animal cells is achieved mainly through modulating SREBP cleavage and HMG-CoA reductase degradation [1], [2]. SREBP belongs to basic helix-loop-helix-leucine zipper (bHLH-Zip) family of transcription factors. The nascent SREBP is targeted to the endoplasmic reticulum (ER) membrane by a hairpin fashion without any transcription activity. In sterols depleted cells, SCAP escorts SREBP to Golgi and SREBP is proteolytically processed by site 1 protease (S1P) and site 2 protease (S2P). Then the NH_2_-terminal bHLH-Zip domain of SREBP is released from membrane and enter nucleus, where it acts as a transcription factor to enhance transcription of genes encoding enzymes in cholesterol and fatty acids biosynthesis pathway [3]. However, high level of sterols especially 25-HC, an analog of cholesterol, blocks the SREBP/SCAP complex translocation. This is due to 25-HC promotes SCAP to bind Inisg-1 or Insig-2, two ER-retention proteins [4]. Thus, the cholesterol synthesis is suppressed.

HMG-CoA reductase,a rate-limiting enzyme of the mevalonate pathway, contains two distinct domains: 1) The NH_2_-terminal transmembrane domain which anchors the reductase on the ER-membrane, and 2) the COOH-terminal soluble catalytic domain which converts HMG-CoA to mevalonate [5]. Previous studies have shown that the membrane domain of HMG-CoA reductase is not only necessary but also sufficient for its sterol-accelerated degradation [6]. High level of sterols promotes the NH_2_-terminal transmembrane domain of HMG-CoA reductase to interact with Insigs. Insigs bridge the interaction between HMG-CoA reductase and gp78, an ER -anchored ubiquitin ligase [7]. Through the cascade reactions performed by ubiquitin-activating enzymes (E1s), ubiquitin-conjugating enzymes (E2s), and ubiquitin ligases (E3s), the reductase was modified by ubiquitin chains mainly at Lys248 [8]. With cooperation with other associated proteins, Ufd1, p97/VCP, and Aup1, the ubiquitinated reductase was retrotranslocated from lipid droplet-associated ER membrane into cytosol for proteasomal degradation [9]–[12].

In the history of studying cholesterol metabolism, 25-HC, which is a hydroxylated derivative of cholesterol, is commonly used. 25-HC not only blocks SREBP processing but also promotes the degradation of HMG-CoA reductase [13], [14]. Thus, 25-HC can block cholesterol synthesis. For mammalian cells cultured in lipoproteins-deficient serum (LPDS), 25-HC is toxic because it cannot be substitute for cholesterol as a structural component in cell membrane. Therefore, 25-HC can be used as a potent lethal selection regent to isolate mutant cell lines with continual synthesis of cholesterol even at the presence of oxysterols [15]. Different from 25-HC, however, another regent SR-12813 can specifically promote HMG-CoA reductase degradation without inhibiting SREBP pathway. Thus, SR-12813 can be used for selection of mutant cells with incapability of accelerating reductase degradation [14].

In past decades, a series of biochemical and genetic experiments have been performed to identify key factors involved in SREBP processing and HMG-CoA reductase degradation pathways [1], [15], [16]. Among which, the somatic cell genetic approach has been proved to be a powerful tool. However, for the most of isolated mutants, which usually are mutagenized chemically (such as EMS, nitrosoethylurea, et.al) or physically (γ-irradiation), it is a challenge to identify the mutant gene(s). Therefore, in the current study, we evaluated the utility of VBIM system to mutagenize animal cells for isolating sterol-resistant or SR-12813-resistant clones. The resultant mutants which survived 25-HC, SL-1, SL-2 and SL-3 contain a truncated NH_2_-terminal domain of SREBP-2 and SL-4 contains overexpressed SCAP. Furthermore, we also isolated one SR-12813-resistant mutant, designated SL-5, which was found to produce a truncated COOH-terminal catalytic domain of HMG-CoA reductase. From the studies on these mutants, the central roles of SREBPs and HMG-CoA reductase in the regulation of lipid homeostasis of animal cells were further verified.

## Materials and Methods

Sterols were from Steraloids, Inc. (Newport, Rhode Island); SR-12813 was from Sigma-Aldrich; lipoprotein-deficient serum (LPDS, d>1.215 g/ml) was prepared from newborn calf serum by ultracentrifugation. Primary antibodies used in this paper for immunoblotting were as follows: mouse monoclonal antibody against the catalytic domain of hamster HMG-CoA reductase (450–887 aa) IgG-A9. Mouse monoclonal antibody against the NH_2_-terminal of hamster SREBP-2 (32–250 aa) IgG-7D4. Mouse monoclonal antibody against the hamster SCAP (540–707 aa) IgG-9D5. Rabbit polyclonal antibody against GFP was generated by immunizing rabbits followed by affinity purification with antigens. Goat polyclonal antibody against ER marker Calnexin (Santa Cruz). Other regents were described previously [17]. The VBIM system, including five plasmids pMD2.G, pCMV-dR8.74 and VBIM-SD-1/2/3 were gifts from Dr. George R. Stark in Cleveland Clinic Foundation.

### Cell culture

CHO-7, HeLa, 293T cells and all mutant cell lines were maintained in monolayer culture at 37°C in a 6% CO_2_ incubator. CHO-7 cells and the mutants derived from it were grown in medium A (1∶1mixture of Ham’s F-12 medium and Dulbecco’s modified Eagle’s medium containing 100 units/ml penicillin and 100 µg/ml streptomycin sulfate) supplemented with 5% (v/v) fetal bovine serum (FBS). 293T cells, HeLa cells and its deriving mutants were grown in medium B (Dulbecco’s modified Eagle’s medium containing 100 units/ml penicillin and 100 µg/ml streptomycin sulfate) containing 10% (v/v) FBS.

### VBIM virus production

One of lentiviral vectors VBIM-SD-1/2/3 were transfected into 293T cells together with second-generation packaging plasmid pMD2.G, pCMV-dR8.74. Supernatants containing virus were collected at 48 and 72 hours were frozen in aliquots. Viral titer was determined before being used.

### Cell mutagenesis and isolation of 25-HC and SR-12813-resistant mutants

On day 0, 1×10^7^ CHO-7 or HeLa cells were set up into medium A contains 5% LPDS or medium B containing 10% LPDS, respectively. On day 1, the cells were infected with VBIM virus with multiplicity of infection (MOI)≈0.3. On day 2, the cells were split and set up into medium containing LPDS with 0.1 µg/ml 25-HC (for CHO-7 cells) or 12 µM SR-12813 (for HeLa cells) at 5×10^5^ per 100 mm dish. Fresh medium was changed per 2 or 3 days. On day 30, the surviving clones were picked up and proliferated.

### Immunoblot analysis

Two dishes of cells were pooled and collected at 1000 g at 4°C for 5 min. Each sample were lysed in 120 µl RIPA buffer (150 mM NaCl, 0.1% SDS, 1.5% NP-40, 0.5% deoxycholate and 2 mM MgCl_2_ in 50 mM Tris-Cl, pH 8.0). The lysed materials were centrifugation at the highest speed at 4°C for 10 min. The 90 µl supernatant was transfered to a new tube and followed by adding 90 µl HMGCR Solubilization buffer (62.5 mM Tris-HCl, pH 6.8, 15% SDS, 8 M Urea, 10% glycerol and 100 mM DTT), after which 4×SDS loading buffer (1% SDS, 6% M 2-Mercaptoethanol, 30% glycerol, 150 mM Tris-Cl, pH 6.8, and trace of Bromophenol Blue) was added to a final concentration of 1×. The samples were incubated in 37°C for 30 min prior to SDS-PAGE. After SDS-PAGE on 8% gels, proteins were transferred to nitrocellulose filters (Amersham). The filters were incubated with the antibodies described in the figure legends.

### Cre recombinase assay

Each mutant cell was set up at 1×10^4^ per well for 12-well plate for double wells on day 0. On day 1, the cells were infected with control adenovirus or adenovirus expressing Cre recombinase (Ad-Cre) (a gift from Dr. Hongbing Ji, SIBS, China). On about day 5 to day 7, when the cells were infected by Ad-Cre and lost their GFP signal, the cells were expanded and characterized.

### Invert-PCR and reverse transcriptase PCR

For invert-PCR, genomic DNA was isolated from cells using Qiagen DNeasy Blood & Tissue Kit. 10 µg DNA was digested with EcoR I and Mfe I for 4 hour at 37°C. The digested DNA was purified with Qiagene MinElute Purification Kit and ligated for 12–16 hour at 16°C. The ligated DNA was purified with Qiagene MinElute Purification Kit and subject to nest-PCR with primers pair (#2/#3) and (#1/#4). The primers sequence was as follows: #1 5′-CCAGAGAGACCCAGTACAAGC-3′, #2 5′-CCAGAGTCACACAACAGACG-3′, #3 5′-GTAAGACCACCGCACAGC-3′, #4 5′-GATCTTCACCTGGAGGAG-3′. For reverse transcriptase PCR, the total RNA was isolated using Trizol (Life technology) according to manufacturer’s instructions and subjected to reverse transcription into complementary DNA (cDNA). The other PCR primers sequences were as follows: P1 5′-CTCTTTGCTCCAGCATGGTCTG-3′, P2, the sequence was same as #1, P3 5′-ATTGCCACGGCGGAACTCA-3′, P4 5′-CATACCAGCAGGCTAAGATGC-3′, P5 5′-CATGGCAGAGCCCACTAAAT-3′.

### Immunofluorescence

The immunofluorescence was performed as previously described [18]. Images were obtained with Leica TCS SP5 laser confocal using an excitatory wavelength of 561 nm and 633 nm for SREBP-2 and Calnexin, respectively.

## Results

**Figure 1:**
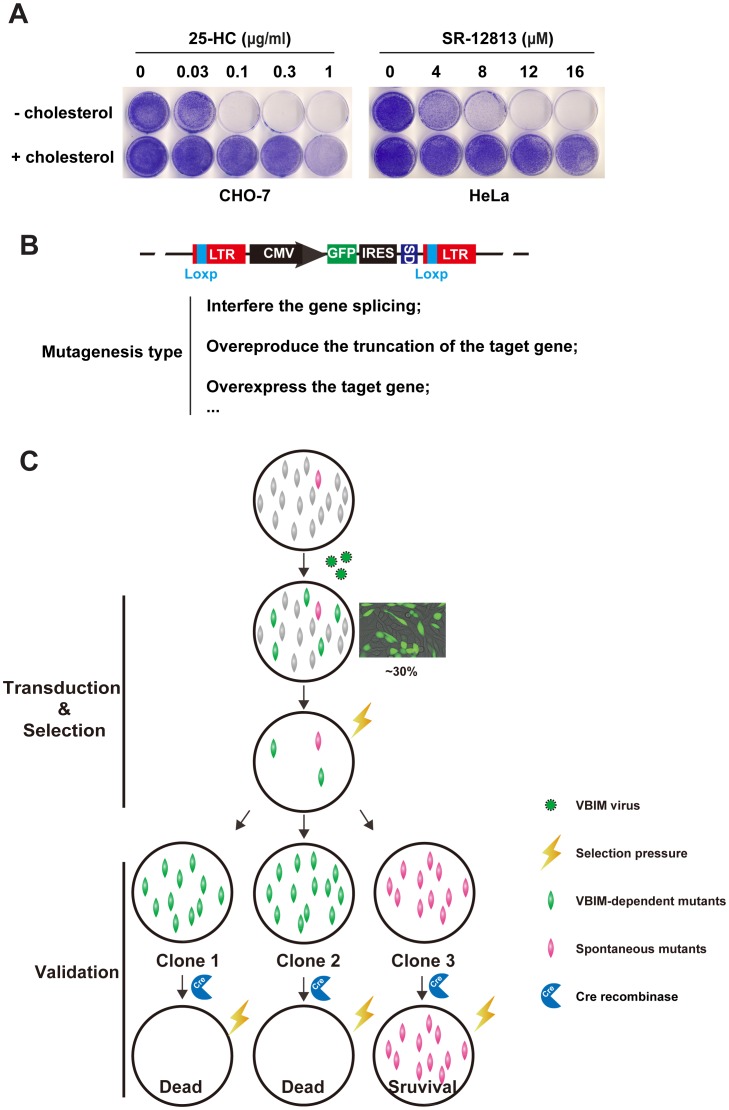
The growth patterns of CHO-7 cells in different selection regents and screen strategy. **A.** On day 0, the CHO-7 cells or HeLa cells were set up at 1×10^4^ per 35 mm dish in medium A contains 5% FBS or medium B containing 10% FBS, respectively. On day 1, the cells were refed with medium A containing 5% LPDS (for CHO-7 cells) or medium B with 10% LPDS (for HeLa cells) with indicated concentration of 25-HC or SR-12813 respectively in the absence (–) or presence (+) of 5 µg/ml cholesterol. The medium were changed every 2 days. On day 14, the cells were washed once with PBS, fixed with 95% ethanol for 20 min and stained with 0.5% crystal violet for 1 hr at room temperature. **B.** The structure of provirus integrating into genome and the mutagenesis type of VBIM virus. LTR, long terminal repeats; CMV, cytomegalovirus promoter; GFP, green fluorescent protein; IRES, internal ribosome entry site; SD, splice donor site; LoxP, Cre-mediated recombination site. **C.** The forward genetic screen strategy. Cell pools were conducted with VBIM virus with MOI≈0.3. The mutagenized cells were selected with 25-HC or SR-12813. The survival clones were isolated, expanded and validated with Cre recombinase.

The experiment of Fig. 1A was designed to determine the minimal concentrations of selecting reagents 25-HC or SR-12813 for killing cells. CHO-7 cells, a clone of CHO-K1 cells that were adapted to growth in lipoprotein-deficient serum and exhibit high rates of cholesterol synthesis [19] could tolerate up to 0.03 µg/ml 25-HC delivered into medium. However, the cells proliferation was completely inhibited at 0.1–1 µg/ml 25-HC, and this phenotype could be restored by addition of 5 µg/ml cholesterol to the medium (Fig. 1A, left panel). Another selecting reagent SR-12813, which belongs to one of 1,1-bisphosphonate esters with hypocholesterolemic activity, was illustrated to have the preference to accelerate the degradation of HMG-CoA reductase [14]. As shown in Fig. 1A (right panel), the SR-12813 killed HeLa cells at concentration range from 8 µM to 16 µM. This toxic effect could also be restored by delivering cholesterol directly to culture medium. These data indicate that both 25-HC and SR-12813 could kill mammalian cells through blocking the synthesis of cholesterol, thereby they are ideal regents for lethal selection.

The VBIM system has been successfully utilized in studying NF-KB signaling pathway [20]. This insertional mutagenesis system was constructed based on lentiviral vector. The “mutagenesis cassette” flanked by two modified long terminal repeats (LTRs) contains the following major components (Fig. 1B): 1) the immediate early cytomegalovirus promoter (CMV), which can drive high-level expression of downstream sequence constitutively in eukaryotic cells, thereby creating dominant mutants; 2) a GFP reporter driven by CMV promoter; 3) an internal ribosome entry site (IRES) and 4) a strong splicing donor (SD) with 3 different reading frames, which allow overexpression of cellular proteins encoded by sequences downstream of insertion site. All the components are flanked by two loxp sites within LTRs, which facilitates to distinguish insertion-dependent clones from spontaneous mutants by removing the “mutagenesis cassette” thoroughly through Cre-LoxP system. The VBIM system can produce several different types of mutagenesis such as encoding truncated proteins, full length proteins, antisense RNA, potentially even microRNA and lncRNA as well as interfering gene splicing, which are dependent on its insertion site and direction in the genome [21].

To identify the crucial regulators in cholesterol synthesis pathway, we conducted a forward genetic screen illustrated in Fig. 1C. 1×10^7^ CHO-7 or HeLa cells were infected by VBIM lentiviral particles at multiplicity of infection (MOI)≈0.3 (the transduction efficient is approximate 30%). The mutagenized cells were subjected to selection with continual 25-HC (for CHO-7 cells) or SR-12813 (for HeLa cells). The selecting reagents-resistant clones were isolated, expanded and validated.

**Figure 2:**
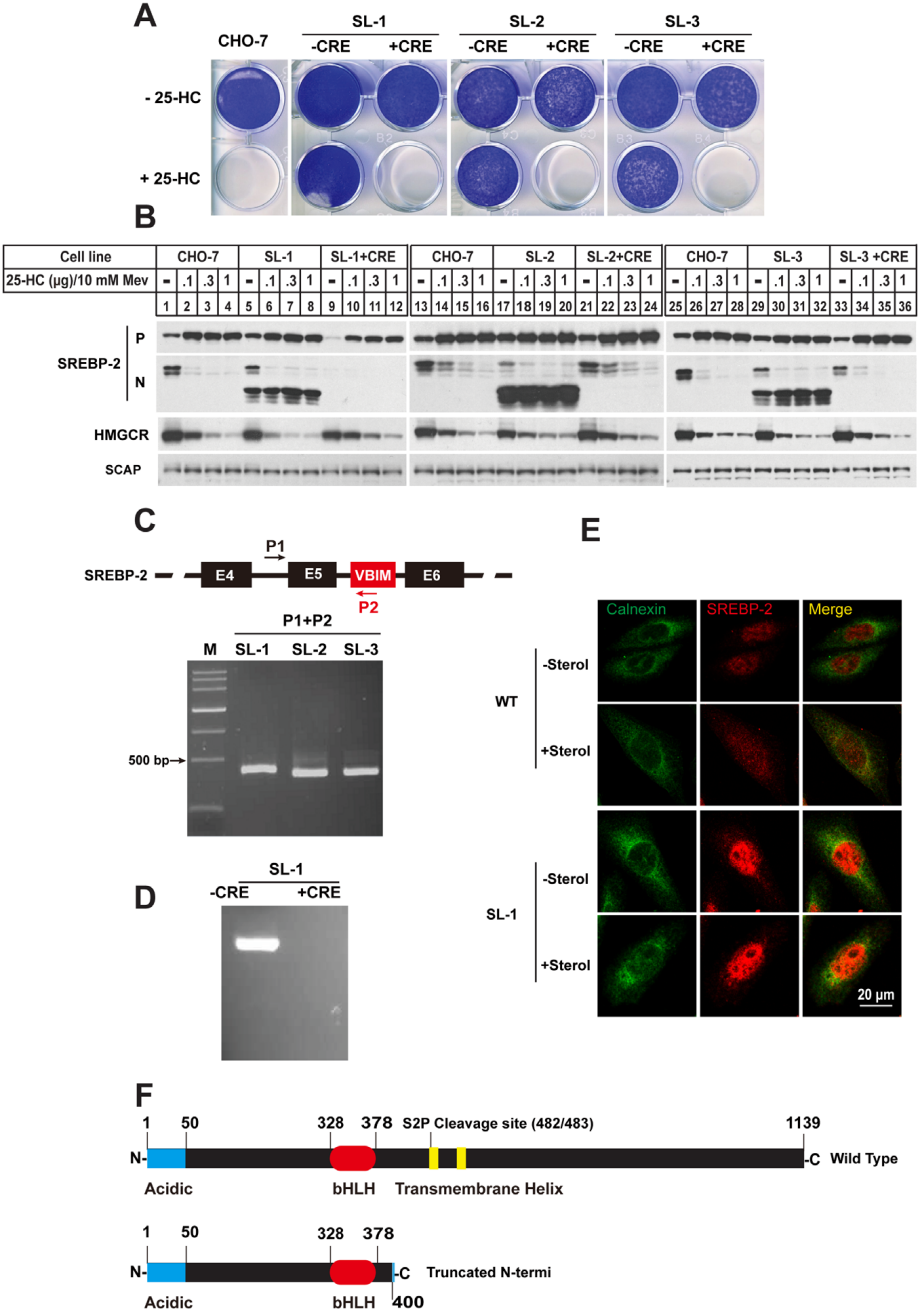
Three 25-HC resistant clones SL-1, SL-2 and SL-3 were identified to contain a truncated SREBP-2. **A.** On day 0, the CHO-7, SL-1, SL-2 and SL-3 and their Cre recombinase processed counterpart (labeling with + CRE) were set up at 1×10^4^ per well for 6-well plate in medium A supplemented with 5% FBS. On day 1, the cells were switched into medium A containing 5% LPDS and 0.1 µg/ml 25-HC. Cells were refed per 2 days. On day 14, the cells were washed once with PBS, fixed with 95% ethanol and stained with 0.5% crystal violet. **B.** CHO-7, SL-1, SL-2 and SL-3 mutants and their Cre recombinase processed counterparts were set up at 4×10^5^ cells per 60 mm dish on day 0 in medium A containing 5% FBS. On day 1 the cells were refed with medium A containing 5% LPDS, 1 µM lovastatin and 10 µM mevalonate. After 16 hr cells were changed to medium A containing 5% LPDS, 1 µM lovastatin and 10 µM mevalonate in the absence (–) or presence of different concentration of 25-HC plus 10 mM mevalonate as indicated. 5 hr post incubation, the cells were harvested as described in “materials and methods”. The aliquots were subjected to SDS-PAGE and immunoblot analysis with IgG-7D4 antibody against SREBP-2, IgG A9 antibody against HMG-CoA reductase and IgG-9D5 against SCAP. P and N denote the precursor and NH2-teriminal mature forms of SREBP-2, respectively. **C.** RT-PCR analysis of three 25-HC resistant clones. The total RNA was isolated and reverse-transcribed into cDNA as described in “materials and methods”. The aliquots of cDNA were subject to PCR analysis with the primers as indicated. The PCR products were analyzed by 1% agarose and purified for sequencing. **D.** Invert-PCR analysis one of three mutants SL-1. The genomic DNA were isolated and carried out invert-PCR analysis as described in “materials and methods”. The PCR products were purified and cloned into pGEM-T easy vector for sequencing. **E.** The localization and regulation of SREBP-2 in WT and SL-1 cells. The fixed cells were stained with polyclonal anti-calnexin antibody and monoclonal anti-SREBP-2 antibody IgG-7D4. **F.** Domain structure of the SREBP-2 of wild type and three sterols-resistance clones. The numbers correspond to the amino acid sequence.

The growth pattern in Fig. 2A showed that three sterol-resistant mutants SL-1, SL-2 and SL-3 can tolerate up to 0.1 µg/ml 25-HC. To validate whether the sterol-resistant phenotype was dependent on VBIM insertion, the mutant cells were infected by adenovirus containing Cre recombinase expression cassette (Ad-Cre). The GFP signal disappeared in cells expressing Ad-Cre, indicating the insertion was excised (Fig. S1). Then growth assay were performed on those cells as well as wild type CHO-7 cells (WT). Different from their counterpart mutant cells, the Cre recombinase treated cells were all killed by 25-HC as wild type cells.

Since 25-HC is a potent inhibitor of SREBP pathway, we then compared the effects of 25-HC on SREBP processing in different cells. As shown in Fig. 2B, the 25-HC inhibited the production of nuclear form of SRBP-2 (N-SREBP-2) in parental CHO-7 cells (lanes 1–4, 13–16 and 25–28) and three mutants (lanes 5–8, 17–20 and 29–32), whereas an obvious lower molecular weight band was detected by IgG-7D4 antibody, which recognizes aa 32 to aa 250 of hamster SREBP-2 in three sterol-resistant mutants (lanes 5–8, 17–20 and 29–32). The truncated N-SREBP-2 failed to response to sterols in all three mutants. When the inserted VBIM were removed by Ad-Cre -mediated excision, the truncated N-SREBP-2 was no longer detected (lanes 9–12, 21–24 and 33–36). Together, these data suggest that the insertion of VBIM virus in these sterol-resistant mutants caused a truncated N-SREBP-2.

To further verify the results, genomic DNA from these mutants was subject to PCR with an upstream primer (P1) that targeted to intron 4 of srebp-2 gene and the downstream primer (P2) which targeted the 5′-end of VBIM virus sequence (Fig. 2C). In the mutant cells, the P1/P2 primer pair gave a band of ∼400 bp, and the size seemed different with slightly variation. The sequencing results of PCR products showed that the VBIM viruses indeed integrated into intron 5 of srebp-2 gene with different sites (Fig. S2). To further confirmed the conclusion from Fig. 2C, the genomic DNA from one mutant SL-1 (–CRE) and its Ad-Cre treated counterpart (+Cre) was conducted by inverse-PCR (iPCR) with both two primers located within the VBIM vector (Fig. S3). The results were shown in Fig. 2D, the mutant SL-1 (–CRE) gave a specific band. The sequencing result of PCR product was consistent with the results of Fig. 2C. Furthermore, the immunofluorescence assay showed that the SREBP-2 signal was mainly in nucleus in WT CHO cells at low sterol condition, indicating SREBP-2 is cleaved and n-SREBP-2 is in nucleus. At high sterol condition, SREBP-2 signal was detected in both nucleus and cytoplasm. However, the SREBP-2 immunofluorescent signal was mainly detected in nucleus of SL-1 mutant cells; no matter the sterol level was high or low (Fig. 2E, bottom panel). Together, these data indicated that the insertion of VBIM virus into intron 5 of SREBP-2 gene produced a truncated N-SREBP-2 which terminated at codon 400. This truncation does not contain transmembrane helix (Fig. 2F), thus it can entry nucleus without sequential cleavages by S1P and S2P proteases.

**Figure 3:**
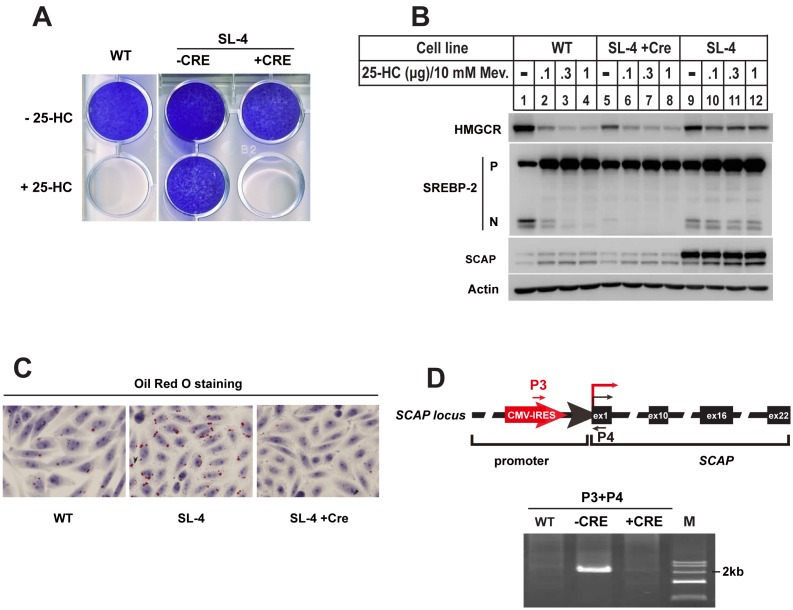
The 25-HC resistant cell line SL-4 contained overexpressed SCAP. **A.** The growth patterns of CHO-7, SL-4 mutant and its Cre recombinase treatment counterpart (SL-4+Cre) in the presence of 25-HC. The cells were set up at 1×10^4^ per well for 6-well plate in medium A containing 5% FBS on day 0. Next day, the cells were changed into the medium containing 5% LPDS plus 0.1 µg/ml 25-HC. Fresh medium was changed every 2 days. On day 14, the cells were washed, fixed with 95% ethanol and stained with crystal violet as descripted in Fig. 2A. **B.** On day 0, the wild type CHO-7, SL-4 and SL-4+Cre cells were set up at 4×10^5^ cells per 60 mm dish in medium A containing 5% FBS. On day 1 the cells were changed to medium A containing 5% LPDS, 1 µM lovastatin and 10 µM mevalonate and incubated at 37°C for 16 hr. Then the cells were switched to medium A containing 5% LPDS, 1 µM lovastatin and 10 µM mevalonate in the absence (–) or presence of different concentration of 25-HC plus 10 mM mevalonate as indicated. 5 hr after treatment, the cells were harvested as described in “**Materials and methods**”. The aliquots were subjected to SDS-PAGE and immunoblot analysis with IgG A9 antibody against HMGCR, IgG-7D4 antibody against SREBP-2, IgG-9D5 against SCAP and anti-actin monomial antibody against Actin. P and N denote the precursor and NH_2_-teriminal mature forms of SREBP-2, respectively. **C.** The neutral lipids of CHO-7, SL-4 and SL-4+Cre cells were visualized by Oil Red O staining. On day 0, the cells were set up at 5×10^4^ cells per well for 12-well plate with coverslips in medium A supplemented with 5% FBS. On day 2, the cells were fixed with 4% PFA, and stained with Oil Red O for lipid droplets (red) and hematoxylin for nuclei (blue). **D.** Genomic PCR analysis for CHO-7, SL-4 and SL-4+Cre cells. Genomic DNA from CHO-7, SL-4 and SL-4+Cre cells were subjected to PCR with primers as indicated. The PCR products were analyzed by 1% agarose and purified for sequencing.

SL-4 is another sterols-resistant mutant clone that we isolated. Fig. 3A showed the growth pattern of CHO-7, SL-4 and its counterparts treated with Ad-Cre (SL-4+Cre). Both CHO-7 and SL-4+Cre cells were killed by 25-HC, but not SL-4. Next, SREBP-2 processing was analyzed by immunoblot (Fig. 3B). When parental CHO-7 cells were incubated with increasing concentrations of 25-HC, the amounts of nuclear form of SREBP-2 declined (N-SREBP-2 of lanes 1–4). The mutant SL-4 cells showed markedly resistance to 25-HC (N-SREBP-2 of lanes 9–12). Furthermore, reductase degradation was no longer sensitive to sterols in mutant SL-4 compared to parental cells (top panel, lanes 9–12 vs. lanes 1–4). However, the mutant cells overexpressing Cre recombinase restored the responds of SREBP-2 and HMG-CoA reductase to sterols (top panel, lanes 9–12 vs. lanes 5–8). Notably, immunoblotting showed that the protein amount of SCAP in SL-4 was much higher than that in CHO-7 cells and SL-4+Cre cells (SCAP, lanes 9–12 vs. lanes 1–4 and 5–8, respectively). Previous studies have shown that excessive synthesis of cholesterol increases the number and size of lipid droplets as form of cholesteryl esters [22]. The Oil Red O staining experiment as shown in Fig. 3C was designed to determine the lipid content in mutant cells. The staining results showed that CHO-7 and SL-4+Cre cells accumulated lipid droplets with small size. However, the size and number of lipid droplets dramatically increased in SL-4 mutant cells. Together, these results suggested that SL-4 cells with overexpressed SCAP result in resistance to sterol regulation on SREBP and HMG-CoA reductase and accumulation of lipids.

We then determined whether the VBIM virus inserted into the upstream of SCAP gene, thereby driving SCAP to be overexpressed. Genomic DNA from wild type CHO-7, mutant SL-4 as well as SL-4+Cre cells were subjected to PCR with primers P3 and P4. The forward primer P3 corresponds to a sequence near to 3′-end of VBIM vector. The reverse primer P4 hybridizes to a sequence near to 5′-end of the first exon of SCAP (top panel, Fig. 3D). The P3/P4 primer pair gave a band of size ∼2 kb in SL-4, but no amplified band in CHO-7 and SL-4+Cre cells (bottom panel, Fig. 3D). The PCR product was sequenced and insertion site was mapped in the SCAP promoter region. These data suggested that the insertion of VBIM into the upstream of SCAP gene conferred the sterol-resistant phenotypes for mutant SL-4.

Previous studies and our screenings have shown that most of the mutants selected by 25-HC are defective in SREBP processing [15]. Therefore, to isolate mutants incapable of sterol-stimulated HMG-CoA reductase degradation, the selecting reagent should specifically promote reductase degradation without suppressing SREBPs proteolysis. It has been reported that a small molecular compound, SR-12813 can satisfy this criterion [14]. Thus we conducted genetic screen in HeLa cells with VBIM, and the SR-12813-resistant clone SL-5 was identified and characterized.

**Figure 4:**
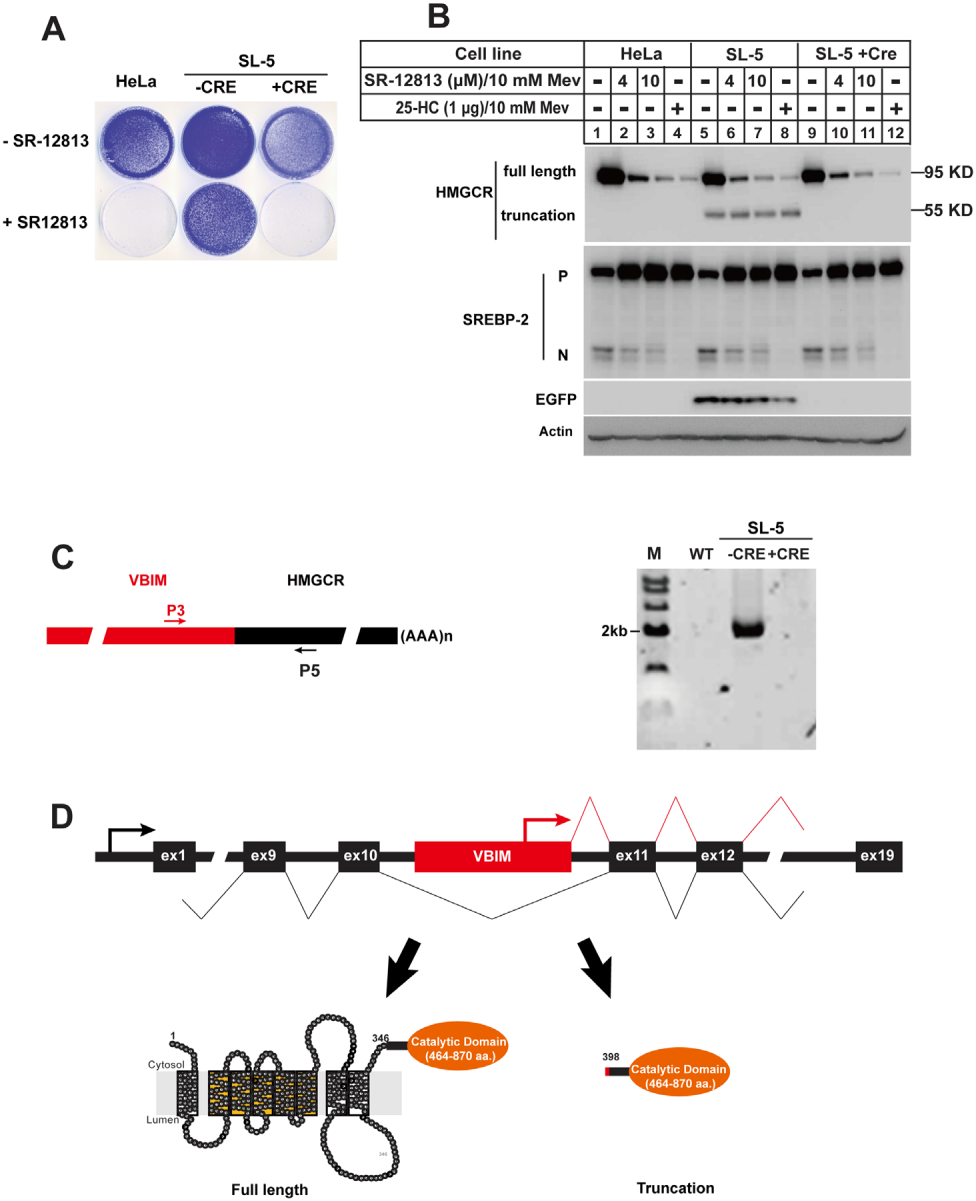
The SR-12813-resistant mutant SL-5 produced a truncated COOH-terminal catalytic domain of HMG-CoA reductase. **A.** Growth pattern of HeLa, SL-5 and its Cre recombinase treatment counterpart (SL-5+Cre). On day 0, the cells were set up in medium B with 10% FBS at 1×10^4^ per well in 6-well plate. On day 1, the cells were changed to medium B with 10% LPDS plus 12 µM SR-12813. The cells were refed every 3 days with fresh medium with SR-12813. On Day 14, the cells were washed, fixed and stained as descripted in Fig. 2A. **B.** The wild type HeLa cells and SR-12813-resistant cell line SL-5 were set up at 4×10^5^ per 60 mm dish in medium B supplemented with 10% FBS on day 0. Next day, the cells were changed to medium B containing 10% LPDS, 1 µM lovastatin and 10 µM mevalonate and incubated for 16 hr. On day 3, the cells were switched to medium B containing 10% LPDS, 1 µM lovastatin and 10 µM mevalonate in the absence (–) or presence of 25-HC or different concentration of SR-12813 plus 10 mM mevalonate as indicated for 5 hr, then the cells were harvested as described in “**Materials and methods**”. The aliquots were subjected to SDS-PAGE and immunoblot analysis. **C.** The total RNA was isolated and reverse-transcribed into cDNA as described in “**Materials and methods**” from the HeLa, SL-5 and SL-5+Cre cells. The aliquots of cDNA were subject to PCR analysis with the primers as indicated. The PCR products were analyzed by 1% agarose and purified for sequencing. **D.** Domain structure of *HMG-CoA reductase* gene and illustration of the insertion site of VBIM virus. The virus inserted into the 10^th^ intron of *HMG-CoA reductase* gene,and the CMV promoter drove transcription of downstream sequences which encoded a truncated COOH-terminal domain of HMG-CoA reductase.

As shown in Fig. 4A, SR-12813 killed wild type cells and mutant cells infected by Ad-Cre (SL-5+Cre), but the mutant SL-5 survived this condition. Next we conducted immunoblot to analysis the degradation pattern of reductase. As shown in Fig. 4B, SR-12813 or 25-HC promoted the degradation of the 95-KDa full-length HMG-CoA reductase in wild type HeLa and SL-5 mutant cells. However, a 55-KDa protein band was detected by reductase-specific monoclonal antibody IgG-A9 (against to COOH-terminal of reductase) in SL-5 mutant (Lanes 5–8). The truncated reductase disappeared after treatment with Ad-Cre (lanes 9–12 vs. lanes 5–8). These results suggested that VBIM insertion created a truncated COOH-terminal of HMG-CoA reductase.

To clarify the accurate integration information of VBIM virus, the reverse transcriptase PCR was conducted in wild type HeLa, SL-5 and SL-5+Cre cells with primers P3 and P5, which are responds to 3′-end VBIM virus sequence and *HMG-CoA reductase* mRNA, respectively (Fig. 4C). The primer pair P3/P5 gave a band with ∼2 kb only in SL-5 mutant. The fusion detail was figured out by sequence results (Fig. S4). The hybrid mRNA infusing part of coding sequence from VBIM vector and *HMG-CoA reductase* mRNA starting with aa 397. Next we performed PCR with genomic DNA of SL-5 with primer P3 and a downstream primer that targets to the sequence near the 5′-end of exon 11, which corresponds to aa 397 of reductase. The sequence results verified the VBIM inserted into intron 10 (data not shown and Fig. 4D). These data demonstrated that the insertion of VBIM produced a COOH-terminal truncated reductase, whose stability is not regulated by sterols.

## Discussion

In this study we describe the isolation and characterization of 5 mutant cell lines resistant to either 25-HC or SR-12813 from forward genetic screen with VBIM system. In the past, most of mutants were mutagenized with chemicals or γ-ray, which does not facilitate the identification of mutant genes. This is why we choose VBIM system to replace the traditional methods. Among the mutants we isolated, SL-1, SL-2 and SL-3, which were isolated with 25-HC from CHO-7 cells, were showed to produce a truncated NH_2_-SREBP-2 that terminated at aa 400, which corresponds to the terminal of exon 5 (Fig. 2). The molecular mechanism is that the excision of intron 5 during *SREBP-2* mRNA maturation is not successful due to VBIM insertion. We speculated that the insertion of such a large size of VBIM backbone (∼5.1 kb) into the small intron 5 (155 bp) interferes the mRNA splicing, thereby producing a truncated N-SREBP-2 (Fig. S2). In fact, when these mutants were treated with Ad-Cre, the VBIM were removed but 238 nucleotides of proviral LTR sequence were left. Theses 238 nucleotides did not to affect mRNA splicing (Fig. 3B).

The phenotypes of SL-1, SL-2 and SL-3 mutants are similar to SRD-1, SRD-2 and SRD-3, three independent mutant cell lines which were isolated before [23]. SRD-1, SRD-2 and SRD-3 contain a truncated N-SREBP-2 terminated at aa 460 which corresponds to 3′-end of exon 6. Theoretically, the truncation at any site between bHLH-Zip domain at residue 400 and the beginning of membrane anchor domain at residue 480 should confer to sterol-resistant phenotypes. However, the isolated mutants in our experiments do not cover all the possibilities. This fact implies that the intron 5 may be a hotspot for integration of lentivirus-derived vectors, or VBIM insertion in the intron 6 (∼3.1 kb) does not interference RNA splicing.

There are four types of sterol-resistant mutants that have been classified so far owning to incapable of suppression of SREBP processing by sterols [15], [22] (Table 1). Type I mutants, including SRD-1, SRD-2 and SRD-3, produce a truncated NH_2_-terminal of SREBP-2 with constitutively activity to enhance the target genes transcription [23]. Apparently, mutants SL-1, SL-2 and SL-3 isolated in our laboratory belong to this subclass, even though these mutants contain a shorter truncated SREBP-2 (Table 1).

**Table 1:** Mutant cell lines with defects in SREBP processing and HMG-CoA reductase degradation*.

Phenotype	Molecular defect	Mutant cell lines	References
**25-HC-resistant** Incapable ofaccelerating reductase degradationin response to sterols and SR-12813	**Type I**--- 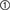 TruncatedSREBP-2 at 460 (dominant)	SRD-1, SRD-2, SRD-3	23
	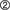 Truncated SREBP-2at 400 (dominant)	SL-1, SL-2, SL-3	This study
	**Type II**---Activating mutationin SCAP (dominant)		
	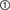 D443N	25-RA, SRD-4, SRD-8	15
	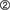 Y298C	SRD-9	15
	**Type III**---DefectiveInsig-1/2 (recessive)	SRD-15	25
	**Type IV**--- 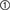 Coupledamplification of SCAP anddefective Insig-1 (dominant)	SRD-19	22
	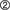 Overexpressed SCAP (dominant)	SL-4	This study
**SR-12813-resistant** Incapable ofsuppressing cholesterolsynthesis in response to sterols	**Type I**---DefectiveInsig-1 (recessive)	SRD-14	14
	**Type II**---Truncated COOH-terminalof HMG-CoA reductase fromaa 397 (dominant)	SL-5	This study

*This table is modified from TABLE II of reference [15].

Type II mutants, including 25-RA, SRD-4, SRD-8 and SRD-9, harbor a dominant active mutation in the putative sterol-sensing domain (SSD) of SCAP, which prevents from binding to Insigs [24]. Therefore, this mutant SCAP can transport SREBPs from ER to Golgi constitutively. Type III mutants belong to a recessive class which has deficiencies of Insig-1 and Insig-2 [25]. Therefore, in these mutant cells, both reductase and SCAP are refractory to sterols. However, our screens did not cover these recessive class mutants.

Type IV mutant contain overexpressed SCAP and Insig-1 deficiency [22]. This mutant is isolated from Insig-1 deficient SRD-14 with higher level of 25-HC. However, our data illustrated that the mutant SL-4 containing overexpressed SCAP is isolated directly from parental CHO-7 cells with 25-HC. Thus, this mutant should be classified into subclass of type IV sterol-resistant mutants. This subclass mutant, together with type II mutants, verifies the central role of SCAP in sterol-regulated SREBP processing.

To obtain the mutants with incapability of accelerating HMG-CoA reductase degradation, SR-12813 has been utilized. For the first time, the SRD-14 mutant cell line with Insig-1 deficient was isolated from CHO-7 cells with SR-12813 [14]. This mutant cell line can be considered as type I mutants with SR-12813-resistance. SRD-14 belongs to a recessive mutant class because it loses the expression of Insig-1. In this paper, we isolated type II SR-12813-resistant mutant SL-5 with a truncated COOH-terminal reductase (Table 1) which conferred a SR-12813-resistant phenotype. SL-5 mutant provides further genetic evidence that the NH_2_-terminal transmembrane domain is required for sterol regulated turnover of reductase.

This study demonstrates that the VBIM system is a powerful and convenient forward genetic tool to identify the regulators of cholesterol metabolism. Unfortunately, our screening presented here does not yield new regulators in SREBP processing or reductase degradation. The possible reasons are that 1) the initial mutagenized cells are not enough to cover all the regulators involved in SREBP processing and reductase degradation pathway and 2) the VBIM system usually generates dominant mutants because of overexpression of RNAs downstream of inserted CMV promoter. Therefore, a recessive mutagenesis system will be developed and applied to discover additional factors.

## Supporting Information

Figure S1 **The GFP signal in SL-1, SL-2 and SL-3 was disappeared after Cre recombinase treratment.** The mutants were treated by adenovirus-mediated Cre recombinase as descripted in “**Materials and methods**”. SL-1, SL-2, SL-3 and their Cre recombinase treated counterparts (+CRE) cell lines were set up at 1×10^5^ per well for 6-well plate in medium A supplemented with 5% FBS. 2 days later, the GFP signal was visualized by microscopy.
(TIF)

Figure S2 **The partial sequence of hamster SREBP-2 and VBIM insertion sites in SL-1, SL-2 and SL-3 mutants.** The black and green nucleotides denote the introns and exons region, respectively. The partial translated amino acids sequence from exon 5 is list below the nucleotides. The red star denotes stop codon. The red triangles indicate insertion sites of VBIM in SL-1, SL-2 and SL-3 mutants.
(TIF)

Figure S3 **Illustration of inverse-PCR for mutant SL-1.**
(TIF)

Figure S4 **Hybrid mRNA and infused protein of VBIM-HMG-CoA reductase in SL-5 mutant cells.** The red nucleotides and amino acids sequence is from VBIM vector.
(TIF)
