# Supplementary figures and images for: Forward Genetic Screening for Regulators Involved in Cholesterol Synthesis Using Validation-Based Insertional Mutagenesis
Source: PLoS One. 2014 Nov 26;9(11):e112632. doi: 10.1371/journal.pone.0112632 (PMC4245081; doi:10.1371/journal.pone.0112632)

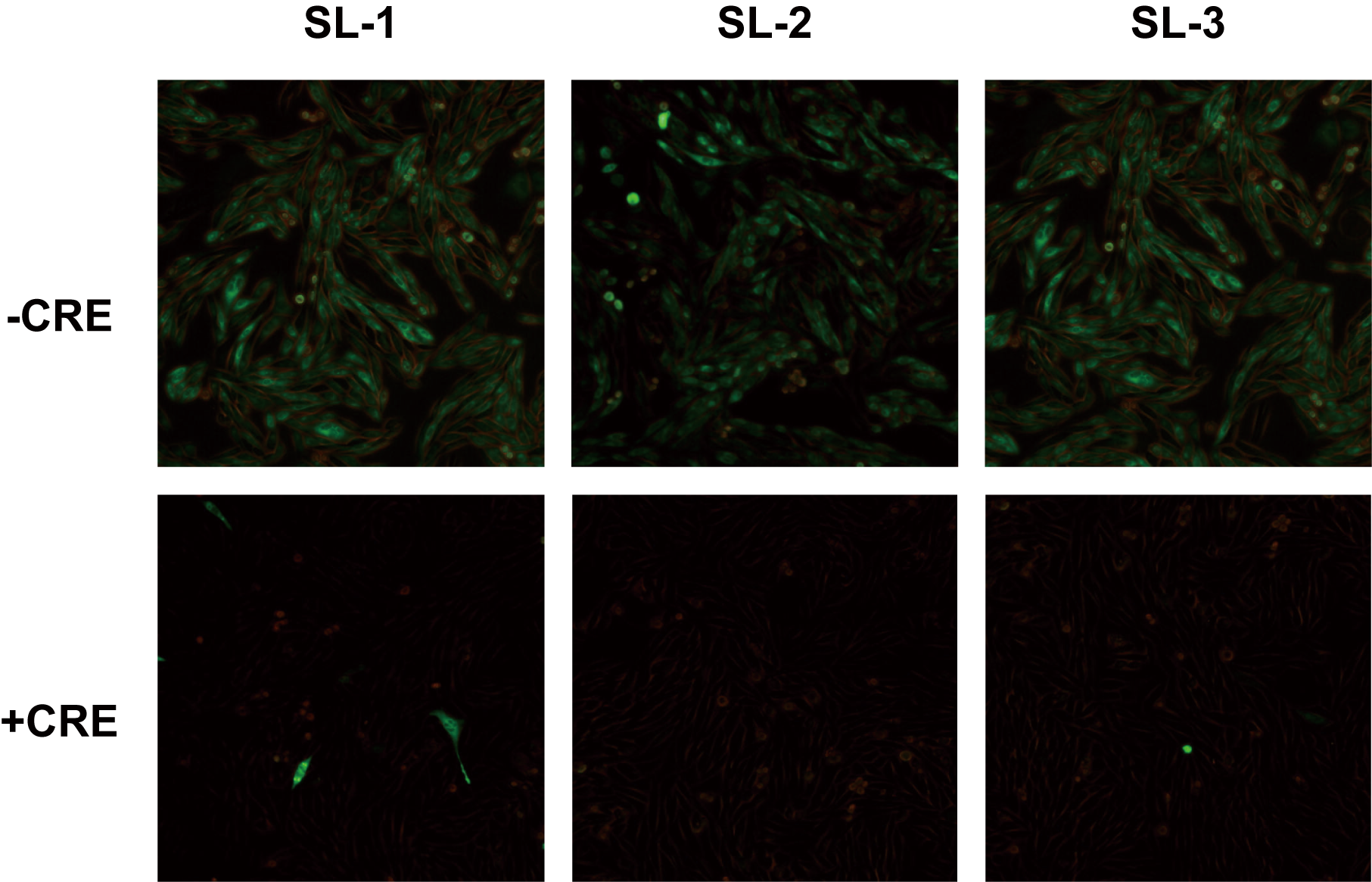

Supplement: Figure S1 — The GFP signal in SL-1, SL-2 and SL-3 was disappeared after Cre recombinase treratment. The mutants were treated by adenovirus-mediated Cre recombinase as descripted in “Materials and methods”. SL-1, SL-2, SL-3 and their Cre recombinase treated counterparts (+CRE) cell lines were set up at 1×10^5^ per well for 6-well plate in medium A supplemented with 5% FBS. 2 days later, the GFP signal was visualized by microscopy. (TIF) [file pone.0112632.s001.tif]

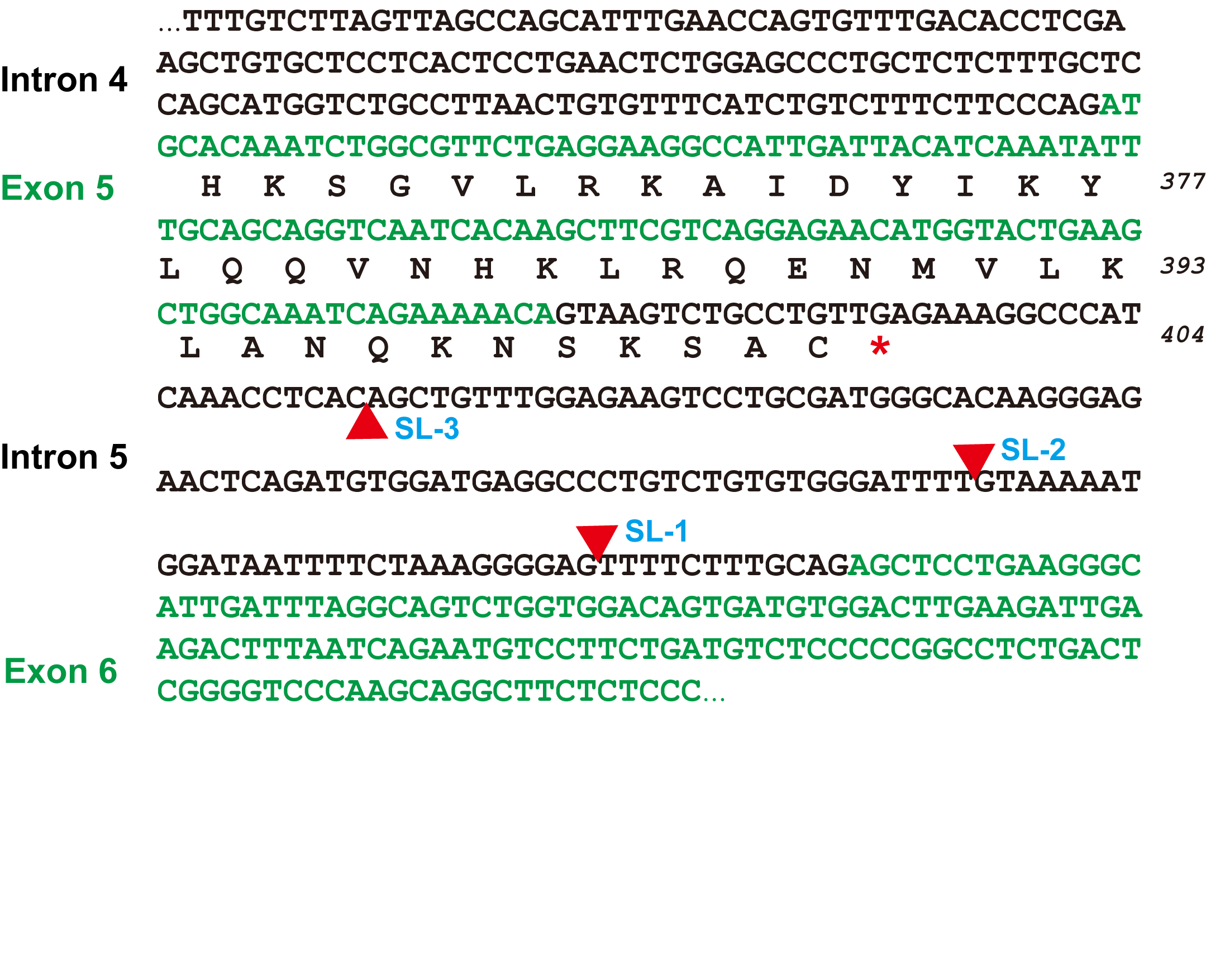

Supplement: Figure S2 — The partial sequence of hamster SREBP-2 and VBIM insertion sites in SL-1, SL-2 and SL-3 mutants. The black and green nucleotides denote the introns and exons region, respectively. The partial translated amino acids sequence from exon 5 is list below the nucleotides. The red star denotes stop codon. The red triangles indicate insertion sites of VBIM in SL-1, SL-2 and SL-3 mutants. (TIF) [file pone.0112632.s002.tif]

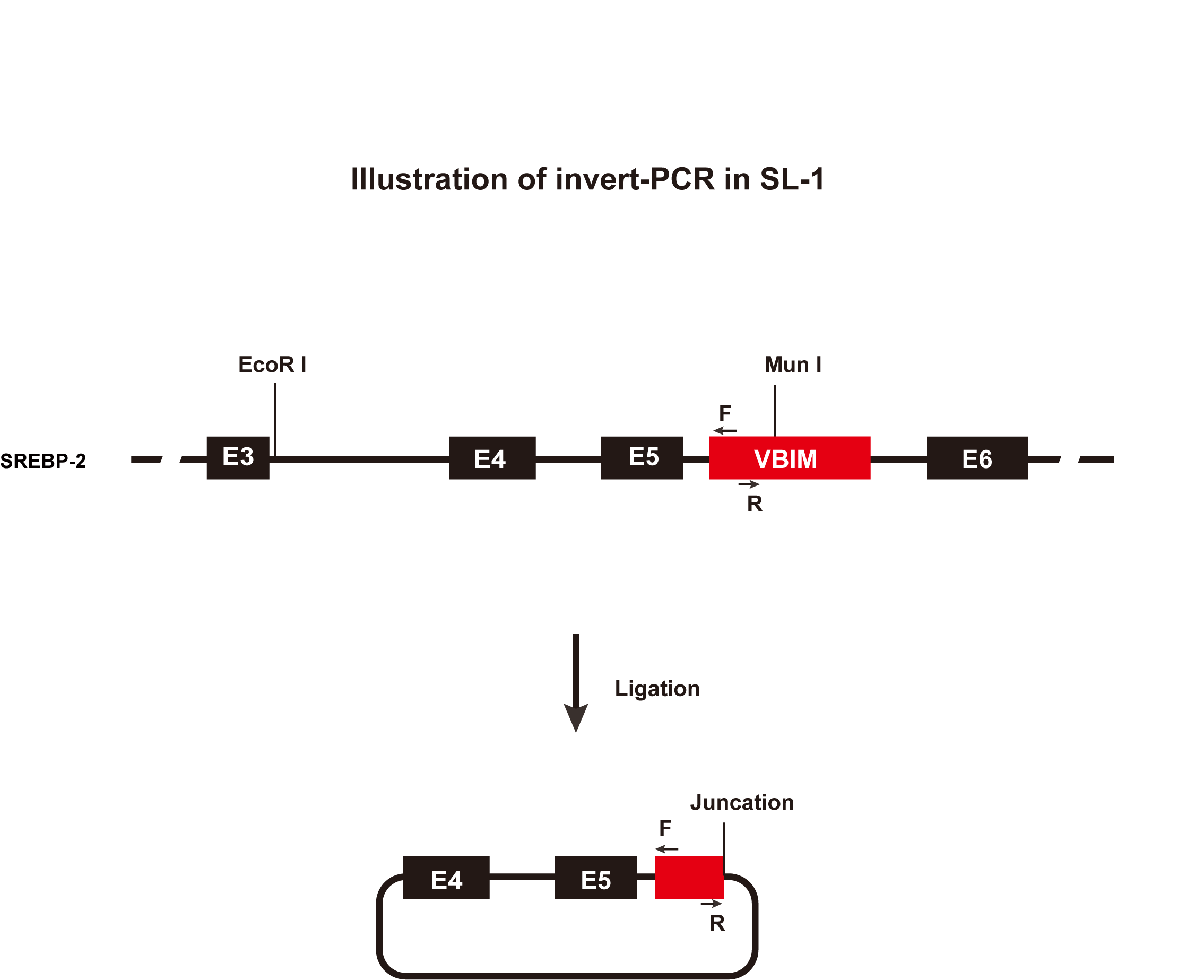

Supplement: Figure S3 — Illustration of inverse-PCR for mutant SL-1. (TIF) [file pone.0112632.s003.tif]

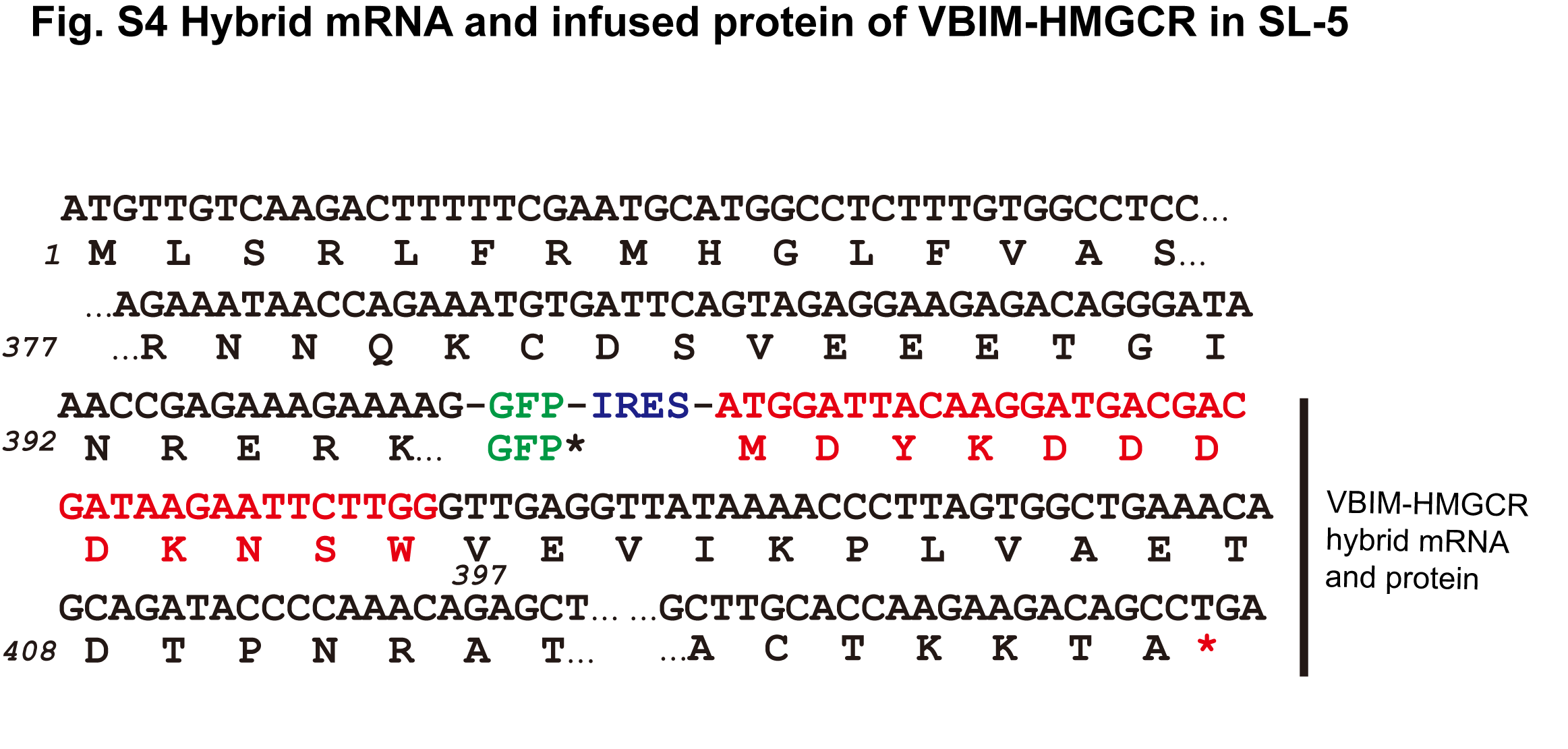

Supplement: Figure S4 — Hybrid mRNA and infused protein of VBIM-HMG-CoA reductase in SL-5 mutant cells. The red nucleotides and amino acids sequence is from VBIM vector. (TIF) [file pone.0112632.s004.tif]
